# A risk score based on procalcitonin for predicting acute kidney injury in COVID‐19 patients

**DOI:** 10.1002/jcla.23805

**Published:** 2021-05-25

**Authors:** Ruo Ran Wang, Min He, Yan Kang

**Affiliations:** ^1^ Department of Critical Care Medicine West China Hospital Sichuan University Chengdu China; ^2^ COVID19 Medical Team (Hubei) of West China Hospital Sichuan University Chengdu China

**Keywords:** acute kidney injury, biomarker, COVID‐19, procalcitonin

## Abstract

**Background:**

Acute kidney injury (AKI) has been reported developing commonly in coronavirus disease 2019 (COVID‐19) patients and could increase the risk of poor outcomes in these patients. We design this study to explore the value of serum procalcitonin (PCT) on predicting AKI and construct risk score for predicting AKI in COVID‐19 patients.

**Methods:**

Patients diagnosed with COVID‐19 and hospitalized in Renmin Hospital of Wuhan University between January 30 and February 24, 2020, were included. The least absolute shrinkage and selection operator (LASSO) regression was performed to identify the strongest predictors of AKI. Multivariate logistic regression analysis was conducted to find independent risk factors for AKI and construct risk score using odds ratio (OR) value of those risk factors. Receiver operating characteristics (ROC) curves were plotted, and area under the ROC curve (AUC) value was calculated to evaluate the predictive value of single PCT level and the constructed risk score.

**Results:**

Among 389 included COVID‐19 patients, 28 (7.2%) patients developed AKI. LASSO regression showed hypertension, saturation of arterial oxygen (SaO_2_), PCT, and blood urea nitrogen (BUN) were the strongest predictors for AKI. After multivariate logistic regression analysis, only SaO_2_ (<0.001), PCT (*p* = 0.004), and BUN (*p *= 0.005) were independently associated with development of AKI in COVID‐19 patients. The AUC of single PCT and constructed risk score was 0. 881 and 0.928, respectively.

**Conclusion:**

PCT level is correlated with AKI in COVID‐19 patients. The efficient risk score consisted of SaO_2_, PCT, and BUN is readily accessible for physicians to evaluate the possibility of AKI in COVID‐19 patients.

## Graphical abstract

Acute kidney injury (AKI) has been reported developing commonly in coronavirus disease 2019 (COVID‐19) patients and could increase the risk of suffering poor outcome in these patients. We design this study to explore the value of serum procalcitonin (PCT) and found that PCT level is correlated with AKI in COVID‐19 patients. The efficient risk score consisted of SaO_2_, PCT and BUN is readily accessible for physicians to evaluate the possibility of AKI in COVID‐19 patients.

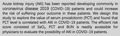

## 1 INTRODUCTION

Having been declared as a global pandemic on 11 March 2020 by the World Health Organization (WHO), the coronavirus disease 2019 (COVID‐19) has brought severe burden to public health and economics overall the world. Up to November 2020, more than 49 million people have been reported infected with SARS‐CoV‐2 and over 1.2 million people unfortunately lost their lives. The cytokine storm developed in COVID‐19 patients, which indicates excessively upregulated production of cytokines, could result in the systemic inflammatory response (SIRS) and subsequent multiorgan dysfunction syndrome (MODS).^1^, ^2^ In addition to the prominent respiratory failure, other organ dysfunction including liver injury, cardiac injury, and kidney injury were also commonly developed in COVID‐19 patients.^3^, ^4^, ^5^, ^6^ Moreover, the development of extra‐pulmonary organ injuries has been confirmed associated with higher mortality of COVID‐19 patients.^7^, ^8^, ^9^, ^10^ Acute kidney injury (AKI), whose incidence ranged from 0.5% to 40% in COVID‐19 patients, could increase the risk of poor outcome and length of hospital stay.^6^, ^11^, ^12^, ^13^, ^14^ Consequently, identifying COVID‐19 patients with high risk of developing AKI during hospitalization and therefore making suitable treatment strategies are essential to improve prognosis of patients.

The procalcitonin (PCT), a precursor of the calcitonin under physiological condition, could be increasingly produced under pathological inflammatory stimulation and is discovered associated with severity of SIRS.^15^, ^16^ Previous studies have found that serum PCT level was useful in predicting the development of AKI during hospitalization for many kinds of diseases including critically ill, traumatic injury, influenza infection, and acute pancreatitis.^17^, ^18^, ^19^, ^20^, ^21^ However, the specific association between PCT and AKI in COVID‐19 patients has not been explored. We designed this study to evaluate the value of PCT on predicting AKI in COVID‐19 patients. Furthermore, we constructed a multivariate risk score to grade the risk level of developing AKI in COVID‐19 patients.

## 2 MATERIALS AND METHODS

### 2.1 Patients

Patients admitted to Renmin Hospital of Wuhan University for COVID‐19 from January 30 to February 24, 2020, were eligible for this study. The diagnosis of COVID‐19 was confirmed by positive results of COVID‐19 RNA in nasopharyngeal swabs utilizing real‐time fluorescence reverse transcription‐polymerase chain reaction (RT‐PCR). Patients transferred from other hospitals and those who lacked clinical or laboratory records were excluded from this study.

### 2.2 Data collection

We collected relevant clinical and laboratory variables from electronic medical record (EMR) system of Renmin Hospital of Wuhan University. Demographic variables (age, sex) and vital signs on admission (mean blood pressure, heart rate, body temperature and respiratory rate, saturation of oxygen) were collected in this study. History of underlying diseases including hypertension, chronic obstructive pulmonary disease (COPD), cardiovascular disease, diabetes mellitus, liver disease, and malignancy were also collected. Variables of blood routine and blood biochemistry tests including serum PCT level were obtained by analyzing blood samples collected at admission. Outcome of this study was the occurrence of AKI in COVID‐19 patients, which was diagnosed based on the Kidney Disease: Improving Global Outcomes (KDIGO) criteria from the second day after admission to discharge. This study was conducted accorded with the Declaration of Helsinki and was approved by the ethics committee of Renmin Hospital of Wuhan University and West China Hospital of Sichuan University. Informed consents of all included patients were signed by patients themselves or their legal representatives.

### 2.3 Statistical analysis

Kolmogorov‐Smirnov test was utilized to confirm the normality of included variables. Normally distributed variables were presented as mean ±standard deviation while non‐normally distributed variables were presented as median (interquartile range). And categorical variables were shown as counts (percentage). We performed Student's t test and Mann‐Whitney U test to compare the difference between two groups of normally distributed variables and two groups of non‐normally distributed variables, respectively. Chi‐square test or Fisher's exact test were conducted to testify the difference between two groups of categorical variables. The least absolute shrinkage and selection operator (LASSO) regression, which could minimize the collinearity of selected risk factors and avoid overfitting of these factors, was performed to identify predictors with nonzero coefficients. Identifying the strongest predictors from plenty of potential risk factors for targeted outcome with relatively small quantity is another advantage of LASSO regression. Predictors with nonzero coefficients were then included in multivariate logistic regression analysis to explore independent risk factors and construct risk score for predicting AKI in COVID‐19 patients. Odds ratio (OR) and 95% confidence intervals (CI) of each risk factors were calculated and presented. The risk score was constructed based on the OR value of each independent risk factor. Receiver operating characteristic (ROC) curves of single predictors and constructed risk score were drawn, and area under the ROC curve (AUC) value of them was calculated. Z test was conducted to verify the difference of AUC between single serum PCT level and the constructed risk score.

Two‐sided P value <0.05 was considered statistically significant. SPSS 22.0 Windows software (SPSS, Inc, Chicago, IL) and R (version 3.6.1; R Foundation) were used to carry out all statistical analyses and figure drawing.

## 3 RESULTS

### 3.1 Baseline characteristics of included COVID‐19 patients

A total of 389 patients confirmed with COVID‐19 were included in this study. Among them, 28 patients developed AKI during their hospitalization with an incidence of 7.8% (Table 1). We divided patients into two groups according to the occurrence of AKI and then compared differences between these two groups. The age of overall patients was 62 (46–71) years. AKI group had significantly higher age than the non‐AKI group (72 vs 60, *p* < 0.001). Sex ratio did not significantly differ between these two groups (57.1% vs 47.1%, *p* = 0.331). Hypertension was the most frequently diagnosed comorbidity which accounted for 23.1% of overall patients. The AKI group had significantly higher percentage of complicated hypertension than the non‐AKI group (60.7% vs 20.2%, *p* < 0.001). Thirty patients were being complicated with COPD, and the percentage of COPD did not differ between two groups with statistical significance (17.9% vs 6.9%, *p* = 0.053), while the AKI group had significantly higher percentage of underlying cardiovascular disease than non‐AKI group (21.4% vs 2.8%, *p* < 0.001). In addition, the percentage of other comorbidities including diabetes mellitus, liver disease, and malignancy did not significantly differ between these two groups (10.7% vs 5.0%, *p* = 0.185; 10.7% vs 3.0%, *p* = 0.071; 3.6% vs 1.7% *p* = 0.410). Records of vital signs at admission showed that AKI group had significantly lower mean blood pressure (99.00 vs 93.33, *p* = 0.045) and saturation of arterial oxygen (SaO_2_) (90% vs 99%, *p* < 0.001) than non‐AKI group. Comparison of laboratory tests presented that white blood cell (9.34 vs 5.64, *p* < 0.001), neutrophil (7.99 vs 3.92, *p*<0.001), AST (56 vs 27, *p* < 0.001), PCT (0.440 vs 0.053, *p* < 0.001), blood urea nitrogen (BUN) (15.40 vs 4.63, *p* < 0.001), serum creatinine (SCr) (160 vs 60, *p* < 0.001), and serum uric acid (SUA) (408 vs 257, *p* < 0.001) were significantly higher in the AKI group while lymphocyte (0.75 vs 1.10, *p* < 0.001), platelet (116 vs 208, *p* < 0.001), and albumin (34.39 vs 37.08, *p* = 0.002) were significantly lower in the AKI group. Considering the drugs usage, AKI group had higher usage percentage of corticosteroids (71.4% vs 21.6%, *p* < 0.001), thymosin (21.4% vs 1.4%, *p* < 0.001), intravenous immunoglobulin therapy (IVIG) (64.3% vs 21.3%, *p* < 0.001), kaletra (35.7% vs 4.2%, *p* < 0.001), arbidol (89.3% vs 67.9%, *p* = 0.010), and interferon (46.4% vs 8.0%, *p* < 0.001) than non‐AKI group. Length of hospital stay did not statistically differ between two groups (22 vs 20, *p* = 0.232). However, the AKI group had statistically higher mortality than non‐AKI group (60.7% vs 16.6%, *p* < 0.001).

**TABLE 1:** Baseline characteristics of included COVID‐19 patients grouped by the development of AKI

Variables	Total (*n* = 389)	Non‐AKI group (*n* = 361, 92.8%)	AKI group (*n* = 28, 7.2%)	*p* value
Age (Years)	62 (46–71)	60 (44–70)	72 (67–84)	<0.001
Sex (Male)	186 (47.8%)	170 (47.1%)	16 (57.1%)	0.331
Comorbidities				
Hypertension (%)	90 (23.1%)	73 (20.2%)	17 (60.7%)	<0.001
COPD (%)	30 (7.7%)	25 (6.9%)	5 (17.9%)	0.053
Cardiovascular disease (%)	16 (4.1%)	10 (2.8%)	6 (21.4%)	<0.001
Diabetes mellitus (%)	21 (5.4%)	18 (5%)	3 (10.7%)	0.185
Liver disease (%)	14 (3.6%)	11 (3%)	3 (10.7%)	0.071
Malignancy (%)	7 (1.8%)	6 (1.7%)	1 (3.6%)	0.410
Vital signs in admission				
Mean blood pressure (mmHg)	93.33 (86.33–98.67)	93.33 (86.33–98.00)	99.00 (88.08–107.00)	0.045
Heart rate (min^−1^)	84 (77–95)	84 (77–96)	81 (76–88)	0.151
Respiratory rate (min^−1^)	20 (18–21)	20 (18–21)	20 (19–32)	0.058
Body temperature (℃)	36.7 (36.5–37)	36.7 (36.5–37)	36.9 (36.4–37.2)	0.562
Saturation of arterial oxygen (%)	99 (98–99)	99 (99–99)	90 (80–99)	<0.001
Laboratory tests in admission				
White blood cell (×10^9^/L)	5.74 (4.23–7.99)	5.64 (4.17–7.83)	9.34 (5.57–12.04)	<0.001
Neutrophil (×10^9^/L)	4.10 (2.58–6.42)	3.92 (2.53–6.18)	7.99 (4.54–10.27)	<0.001
Lymphocyte (×10^9^/L)	1.07 (0.75–1.45)	1.1 (0.77–1.48)	0.75 (0.54–1.07)	<0.001
Monocyte (×10^9^/L)	0.43 (0.29–0.59)	0.43 (0.29–0.59)	0.48 (0.22–0.61)	0.866
Platelet (×10^9^/L)	203 (151–260)	208 (157–262)	116 (93–197)	<0.001
Hemoglobin (g/L)	127 (115–138)	127 (115–137)	135 (121–145)	0.111
Albumin (g/L)	36.88 ±4.73	37.08 ±4.74	34.39 ±3.95	0.002
Globin (g/L)	24.0 (21.7–27.3)	23.8 (21.7–27.2)	25.1 (21.9–28.1)	0.428
ALT (U/L)	25 (16–41)	24 (16–40)	26 (20–52)	0.226
AST (U/L)	28 (20–42)	27 (20–39)	56 (42–76)	<0.001
ALP (U/L)	63 (52–81)	63 (51–80)	68 (55–89)	0.310
Procalcitonin (ng/ml)	0.057 (0.034–0.128)	0.053 (0.033–0.114)	0.440 (0.133–2.433)	<0.001
Blood urea nitrogen (mmol/L)	4.80 (3.70–7.09)	4.63 (3.65–6.60)	15.40 (6.50–30.65)	<0.001
Serum creatinine (umol/L)	61 (50–75)	60 (49–73)	160 (65–293)	<0.001
Serum uric acid (umol/L)	259 (203–343)	257 (198–334)	408 (235–670)	<0.001
Drugs treatment				
Corticosteroids	98 (25.2%)	78 (21.6%)	20 (71.4%)	<0.001
Thymosin	11 (2.8%)	5 (1.4%)	6 (21.4%)	<0.001
IVIG	95 (24.4%)	77 (21.3%)	18 (64.3%)	<0.001
Kaletra	25 (6.4%)	15 (4.2%)	10 (35.7%)	<0.001
Arbidol	270 (69.4%)	245 (67.9%)	25 (89.3%)	0.010
Interferon	42 (10.8%)	29 (8.0%)	13 (46.4%)	<0.001
Ribavirin	32 (8.2%)	29 (8.0%)	3 (10.7%)	0.493
Traditional Chinese medicine	237 (60.9%)	216 (59.8%)	21 (75.0%)	0.103
Length of hospital stay (days)	20 (15–27)	20 (15–27)	22 (12 −37)	0.232
In‐hospital mortality (%)	77 (19.8%)	60 (16.6%)	17 (60.7%)	<0.001

Abbreviations: AKI, acute kidney injury; COPD, chronic obstructive pulmonary disease; ALT, alanine aminotransferase; AST, aspartate aminotransferase; ALP, alkaline phosphatase; IVIG, intravenous immunoglobulin therapy.

### 3.2 Risk factors for AKI in COVID‐19 patients and their predictive value

**FIGURE 1:**
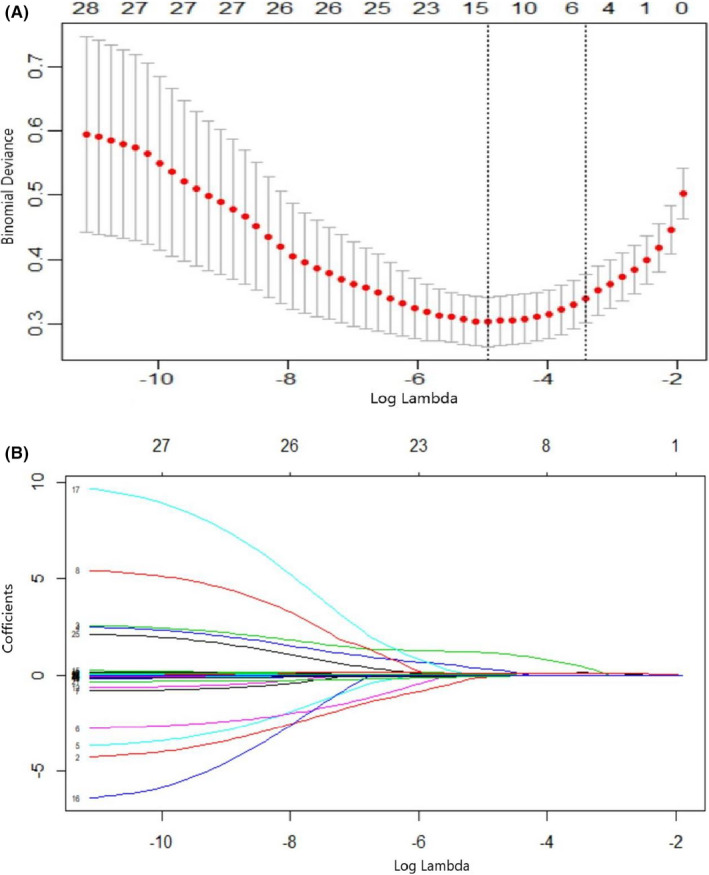
A, The risk predictors selection using the least absolute shrinkage and selection operator (LASSO) binary logistic regression analysis. Two dotted vertical lines mark the optimal values by minimum criteria and 1‐s.e. criteria. Four variables were selected by LASSO binary logistic regression analysis B. LASSO coefficient profiles of the 28 variables

**FIGURE 2:**
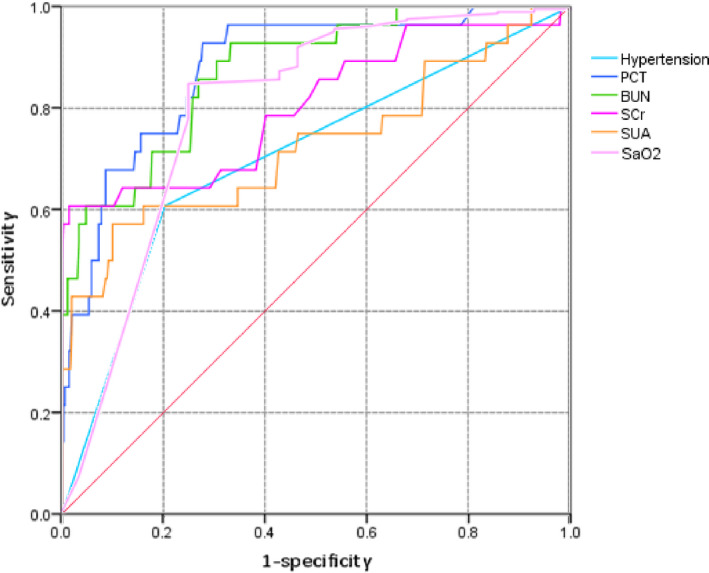
Receiver operating characteristic curve of single PCT and other markers for predicting AKI in included COVID‐19 patients. The AUC value of hypertension, single PCT value, and BUN value was 0.702 (0.593–0.812), 0.881 (0.817–0.945), and 0.874 (0.808–0.940), respectively. And the AUC value of SUA, SCr and SaO_2_ was 0.806 (0.606–0.847), 0.726 (0.702–0.909), and 0.797 (0.693–0.901), respectively

Of 28 characteristics were initially included in LASSO binary logistic regression, and only 4 candidate variables significantly associated with AKI were finally selected including hypertension, SaO_2_, PCT, and BUN (Figure 1A). The weight for these four factors was calculated when log(λ) = −3.0342 and λ = 0.0481 from LASSO binary logistic regression model (Figure 1B). Coefficients for these factors were shown as follows: 0.0043 for hypertension, −0.0181 for SaO_2_, and 0.0880 for PCT, 0.1039 for BUN. ROC curves were plotted to evaluate the predictive value of these four factors and other renal function indicators including SCr and SUA (Figure 2.). The AUC value of hypertension, single PCT value, and BUN value was 0.702, 0.881, and 0.874, respectively (Table 2). And the AUC value of SUA, SCr, and SaO_2_ was 0.806, 0.726, and 0.797, respectively. The specificity, sensitivity, and cutoff value are also shown in Table 2.

**TABLE 2:** The value of single laboratory indicators for predicting AKI in COVID‐19 patients

	AUC	95% Cl	Cutoff value	Sensitivity	Specificity
Hypertension	0.702	0.593–0.812	‐	0.607	0.798
PCT	0.881	0.817–0.945	0.1045	0.929	0.723
BUN	0.874	0.808–0.940	5.565	0.919	0.668
SCr	0.806	0.702–0.909	122.5	0.607	0.986
SUA	0.726	0.606–0.847	401	0.571	0.900
SaO_2_	0.797	0.693–0.901	97.5	0.848	0.750

Abbreviations: AKI, acute kidney injury; AUC, area under the receiver operating characteristic curve; PCT, procalcitonin; BUN, blood urea nitrogen; SCr serum creatinine; SUA serum uric acid; SaO_2_, saturation of arterial oxygen.

### 3.3 The risk score for predicting AKI in COVID‐19 patients constructed by multivariate logistic regression

**FIGURE 3:**
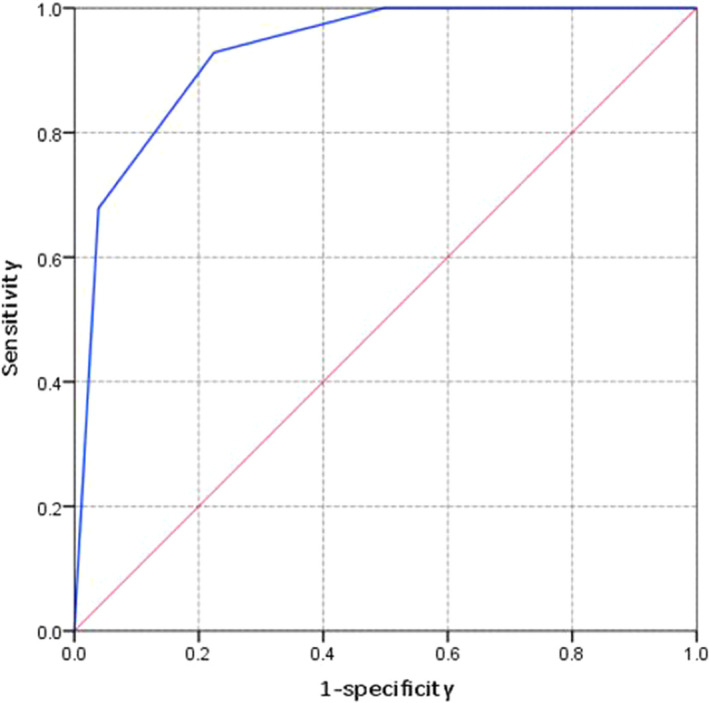
Receiver operating characteristic curve of constructed risk score for predicting AKI in included COVID‐19 patients. The AUC value of this risk score was 0.928

**FIGURE 4:**
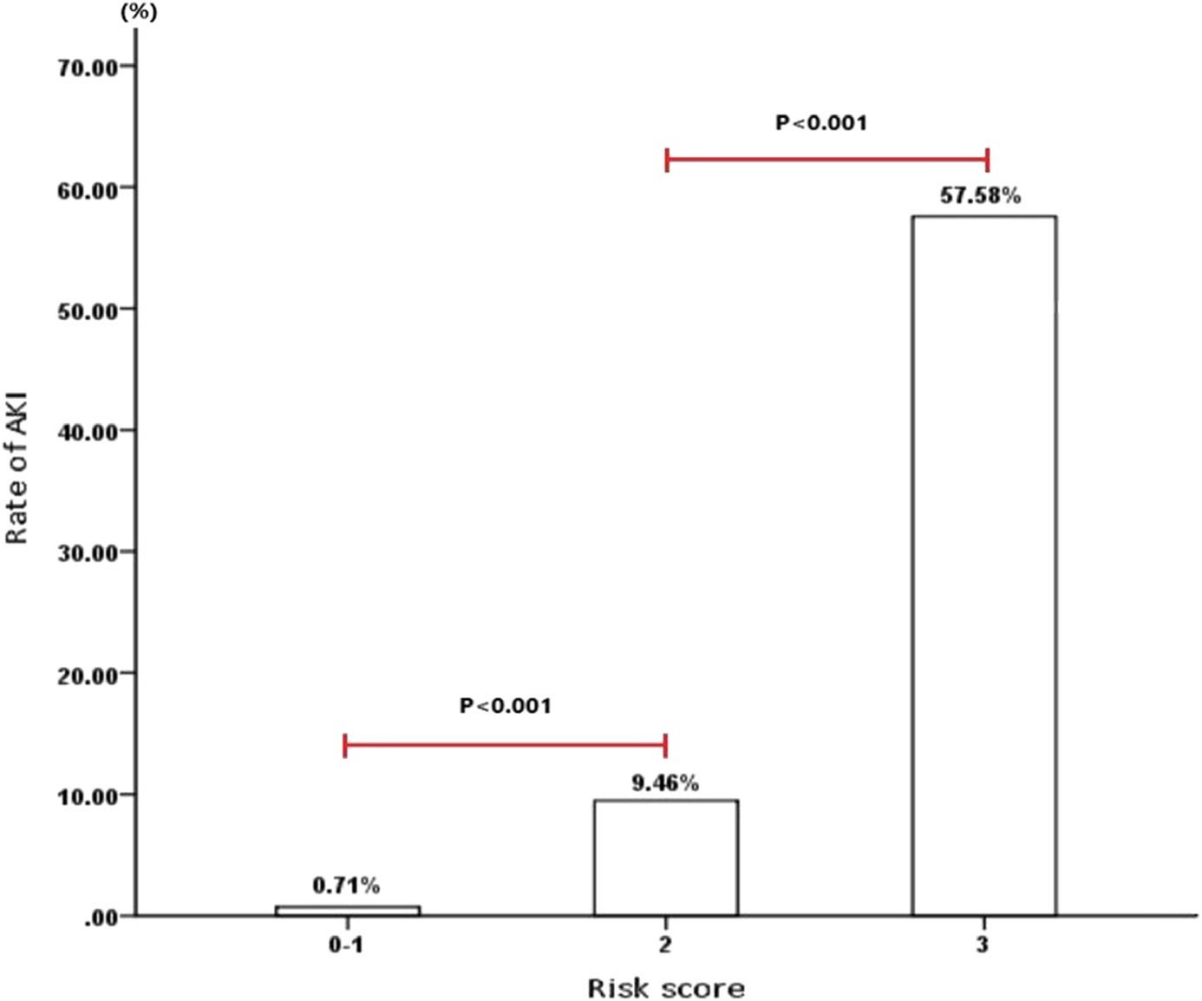
Patients's percentage of AKI grouped by constructed risk score. The incidence of AKI in low‐risk group, medium‐risk group and high‐risk group was 0.71% (2/282), 9.46% (7/74) and 57.58% (19/33), respectively

SaO_2_, PCT, and BUN were transformed into binary variables based on their corresponding cutoff value, Then, SaO_2_, PCT, and BUN remained independent risk factors for AKI in multivariate logistic regression analysis and OR of these three variables are shown in Table 3. Corresponding scores of these three factors were assigned according to their β coefficients. Presented as Table 4, the risk score was readily used in clinical practice, which ranged from 0 to 3. The ROC curve indicated that AUC value of this risk score was 0.928 (95 Cl%: 0.888–0.968) with a sensitivity of 0.888 and specificity of 0.968 (Figure 3). The AUC value of constructed risk score was higher than that of singe PCT value though without statistical significance (Z = 1.2016, *p*>0.05). Among included COVID‐19 patients, 2 patients developed AKI in low‐risk group with AKI incidence of 0.71% (2/282) (Figure 4.). 7 and 19 patients developed AKI in medium‐risk group and high‐risk group with an incidence of 9.46% (7/74) and 57.58% (19/33), respectively.

**TABLE 3:** Multivariate logistic regression analysis of risk factors associated with AKI in COVID‐19 patients

	OR	95% Cl	β coefficient	*p* value
Hypertension				
No	1 [Reference]			0.546
Yes	1.378	0.487–3.901	0.321	
SaO_2_				
≥97.5	1 [Reference]			<0.001
<97.5	9.495	3.373–26.732	2.251	
PCT				
<0.105	1 [Reference]			0.004
≥0.105	9.725	2.040–46.356	2.275	
BUN				
<5.565	1 [Reference]			0.005
≥5.565	9.469	1.964–45.649	2.248	

Abbreviations: AKI, acute kidney injury; OR, odds ratio; Cl, confidence interval; SaO_2_, saturation of arterial oxygen; PCT, procalcitonin; BUN, blood urea nitrogen.

**TABLE 4:** Constructed risk score for predicting AKI in COVID‐19 patients

Variables	Score	Risk levels
SaO_2_		Low risk: Total score<2 Medium risk: Total score=2 High risk: Total score=3
≥97.5	0	
<97.5	1	
PCT		
<0.1045	0	
≥0.1045	1	
BUN		
<5.565	0	
≥5.565	1	

Abbreviations: AKI, acute kidney injury; SaO_2_, saturation of arterial oxygen; PCT, procalcitonin; BUN, blood urea nitrogen.

## 4 DISCUSSION

AKI is a kind of common organ dysfunction which has been observed occurring in 8.4% to 19.6% hospitalized patients.^22^ The incidence of AKI among COVID‐19 patients has been reported ranging from 0.5% to 40% according to different studies. A total of 28 patients developed AKI during hospitalization in our study with incidence rate of 7.20%. A recent meta‐analysis concluded that the pooled AKI incidence of Chinese COVID‐19 cohorts was 5.5% while that reported from the USA and Europe was 28.6%.^14^ This significant variation may be attributable to the different guideline recommendations for hospital admission. In China, both suspected and confirmed cases are required to be effectively isolated and received treatments in hospitals, while American hospitals only accept severely ill patients with obvious symptoms, who actually have older age and more underlying diseases than Chinese patients. Increased age and comorbidities including diabetes, hypertension, cardiovascular disease, and liver disease all have been confirmed as risk factors for AKI development and mortality of COVID‐19 patients.^6^, ^23^, ^24^ Consistent with previous studies, our study showed AKI group of included COVID‐19 patients had significantly older age and higher incidence of complicated hypertension and cardiovascular disease than non‐AKI group. The fact that mortality of AKI group was higher than that of non‐AKI group also indicated AKI development was unfavorable to outcome of COVID‐19 patients and suitable treatment decisions should be taken to avoid this detrimental complication.

The mechanism involved in the development of AKI in COVID‐19 patients may be multifactorial. Bioinformatic analyses indicated that angiotensin‐converting enzyme 2 (ACE2), which is acknowledged as a major receptor of SARS‐CoV‐2, is highly expressed in renal tissue.^25^, ^26^ Kidney specimens obtained from autopsy of patients with COVID‐19 presented proximal tubular lesion accompanied with loss of brush border and vacuole degeneration in light microscopy. In addition, clusters of typical spike‐like nucleocapsid proteins were observed in the tubular epithelium and podocytes by electrical microscopy and prominent ACE2 expression was found in proximal tubule by immunohistochemical staining.^27^, ^28^ These evidences suggested SARS‐CoV‐2 could produce cytotoxicity effects on renal cells and therefore result in renal dysfunction by directly binding to ACE2 of renal tubular cells. The cytokine storms, a characteristic pathophysiological process in COVID‐19 infection, may also play an important role in the development of AKI. The invasion and reproduction of virus could over‐activate human immune system with subsequent massive release of cytokines including interleukin‐2R (IL‐2R), interleukin‐6 (IL‐6), interleukin‐7 (IL‐7), interleukin‐10 (IL‐10), tumor necrosis factor‐α (TNF‐α), granulocyte‐macrophage colony‐stimulating factor (GM‐CSF), monocyte chemoattractant protein‐1 (MCP‐1), and macrophage inflammatory protein‐1A (MIP‐1A).^29^, ^30^, ^31^ Previous studies have confirmed that proinflammatory cytokines, especially the IL‐6, play a key role in the pathophysiological process of AKI.^32^, ^33^, ^34^ Besides, other common risk factors during viral infection including hypotension, hypoxemia, thrombosis, rhabdomyolysis, and unsuitable use of nephrotoxic drugs may also collectively aggravate the abnormal renal function.^28^, ^35^, ^36^

In this study, multivariate logistic regression analysis showed that SaO_2_, PCT, and BUN were three independent risk factors for AKI in COVID‐19 patients. The hypoxemia, which sometimes be defined as SaO_2_ <90%, has been verified associated with deteriorating renal function.^37^, ^38^ A recent study showed COVID‐19 patients with AKI whose renal function improved had higher oxygenation index than those did not improved. Therefore, it is logical and effective to include SaO_2_ into our constructed risk score. Another constituent in our risk score is PCT, which has been confirmed as a reliable maker for predicting AKI in many kinds of patients including those diagnosed with critically ill, traumatic injury, influenza infection or acute pancreatitis.^17^, ^18^, ^19^, ^20^, ^21^ In our study, the single PCT level indeed had high predictive value with AUC of 0.881. The close correlation between PCT and AKI in COVID‐19 patients may be mediated by the systemic inflammation. Previous studies found that serum PCT level could increase in patients with systemic inflammation response syndrome (SIRS) and proinflammatory cytokines including TNF‐α and IL‐6 could upregulate the expression of PCT mRNA in peripheral monocytes.^39^, ^40^ The increased PCT could in turn stimulate the mesangial cell to upregulate the production of TNF‐α and IL‐6.^41^ Experimental studies even found that immunoneutralization of PCT could alleviate renal injury in porcine model of sepsis.^42^, ^43^ As a crucial component of systemic inflammation cascade, PCT expression may indicate the severity of systemic inflammation and therefore correlated with the AKI development in COVID‐19 patients. Consisted of SaO_2_, PCT, and BUN, our risk score comprehensively reflecting the effect of hypoxemia and systemic inflammation on renal function, is readily available and effective on predicting AKI in COVID‐19 patients.

Our study had several limitations. Firstly, this study was performed in a single medical center. And a few of patients lacking records of PCT level on admission were excluded from this study so that the selection bias could not be avoided. Secondly, urine volume, an important indicator for diagnosis of AKI, was not recorded in our study. This is partly due to the lack of records of urine volume in mildly and moderately ill patients. Thirdly, the detailed extent of lung involvement was not recorded by us which may affect the severity of kidney injury. Finally, we did not divide overall enrolled patients into training set and validation set due to the limited number of AKI event. Further study with larger sample size could be conducted in other medical centers to verify the value of our risk score.

## 5 CONCLUSIONS

Single PCT value is a valuable predictive marker of AKI in COVID‐19 patients, the risk score we constructed using serum PCT level could readily and efficiently help clinicians evaluate the possibility of developing AKI in COVID‐19 patients.

## CONFLICT OF INTEREST

All authors declare no conflict of interest.

## AUTHOR'S CONTRIBUTIONS

Ruoran Wang was involved in the data analysis, and drafting of the manuscript. Min He was involved in the data collection and interpretation of the results. Yan Kang interpreted the results and contributed to the reviewing of the draft.

## Data Availability

The data used and analyzed during this study are available from the corresponding author on reasonable request.
